# Implementing Smartphone Tutorials for Patients With Severe Glaucoma

**DOI:** 10.7759/cureus.39537

**Published:** 2023-05-26

**Authors:** Nathaniel Cameron, Akhila Alapati, Megan Haghnegahdar, Harrison Sciulli, Jordan Miller, Jacob O’Dell, William Bray

**Affiliations:** 1 Department of Ophthalmology, University of Kansas School of Medicine, Kansas City, USA; 2 Department of Ophthalmology, University of Kansas Medical Center, Kansas City, USA; 3 Department of Surgery, University of Kansas Medical Center, Kansas City, USA; 4 Department of Ophthalmology, Kansas City Veterans Affairs (VA) Medical Center, Kansas City, USA

**Keywords:** primary open-angle glaucoma, patient safety-based medical education, glaucoma practice, video-based learning, cellular phone

## Abstract

Purpose

The purpose of the study is to determine if instructional videos detailing the use of smartphone accessibility features may be used to improve quality of life and comfort with phone usage among patients with severe glaucoma.

Design

The design of the present study is an interventional case series.

Methods

The patients with vision loss due to severe glaucoma were recruited from one institution. Two surveys were completed to provide baseline data: one detailed their current use of smartphone accessibility features, and the other provided survey was the EuroQol 5 Dimension 5 Level (EQ-5D-5L) (EuroQol Group, Rotterdam, Netherlands), which is used to assess the quality of life. Then, the patients were shown a brief video with instructions on configuring the use of voice-over, magnification, and zoom functions, along with other features. To conclude, the patients completed the same surveys either at follow-up visits or by phone calls.

Results

Fifteen patients were recruited to participate in the study. At baseline, the participants used a median of one accessibility feature, with the most common feature being “text sizing/bolding.” At follow-up, the participants averaged the gain of use of one accessibility feature and reported a decrease in text messaging visual limitation, although these findings did not reach statistical significance. Overall, the quality of life, as measured by the EQ-5D-5L, demonstrated a non-statistically significant increase of six points.

Conclusions

Despite the lack of statistical significance, our results indicate that providing instructional videos may benefit the patients’ ability to navigate on their smartphones. Incorporating links or Quick Response (QR) codes to these instructional videos provides an opportunity to improve the quality of life at no additional risk to the patient. Further studies are needed with an increased population to investigate for any significance of our findings.

## Introduction

Current smartphones are equipped with accessibility features for low-vision users. Recent surveys have demonstrated significant interest among the low-vision population in using features found on mainstream devices, such as tablets and phones [1]. Despite patient interest, there are important barriers to incorporating smartphone accessibility features into a daily routine. Principally, there is a lack of definite education on navigating the use of these tools. For example, one review found that only 12% of the patients using smartphone accessibility tools received clinical instruction on their use, and the majority taught themselves how to install the features [2]. Increased adoption of low-vision features on mainstream devices also combats financial barriers posed by more traditional assistive devices, such as electronic magnification devices and digital accessible information system (DAISY) readers [3].

Though smartphone visual aid features can benefit patients with various etiologies of vision loss, it is possible that their use can uniquely serve glaucoma patients. Despite being a prominent cause of low vision in the United States, one review found that glaucoma patients make up just 14% of patients receiving low-vision services [4]. This finding is surprising given the documented difficulty in dark adaptation, reading, and activities of daily living in patients with glaucoma [5-7]. Improving the understanding of smartphone accessibility tools represents an opportunity to improve overall care and quality of life for patients with glaucoma.

In this study, we aim to address the lack of direct, clinical education on navigating accessibility features found in smartphones. Our principal objective was to determine if instructional videos detailing the setup of smartphone accessibility features would impact the quality of life measures and phone usage among patients with severe glaucoma.

## Materials and methods

This study was approved by the University of Kansas Medical Center (KUMC) Institutional Review Board. The patients seen at the University of Kansas (KU) Eye Center’s Glaucoma Clinic from January 2022 to June 2022 were recruited for participation. The inclusion criteria were patients diagnosed with advanced glaucoma, as defined by a glaucomatous optic disc with visual field loss in both hemifields and/or a defect within five degrees of fixation [8]. The patients with severe glaucoma in one or both eyes from all etiologies were included. The patients with vision loss due to other conditions were excluded from the study. Each recruited patient signed an informed consent form detailing the risks and benefits of their participation after a thorough explanation of the study was given to them.

The patients completed two surveys to record baseline values. The first survey was designed to measure their initial usage of smartphone accessibility features and perceived visual limitations on using their phone for communication. The participants then completed the EuroQol 5 Dimension 5 Level (EQ-5D-5L) survey, a survey created by the EuroQol Group (Rotterdam, Netherlands) to assess health-related quality of life in the participants, specifically by examining five dimensions: mobility, self-care, usual activities, pain/discomfort, and anxiety/depression [9]. The participants may then choose from five levels of responses, ranging from no issue in the specified domain to extreme difficulty. The EQ-5D-5L survey has been validated in various studies looking at quality-of-life measures in patients with various medical conditions [10,11].

After recording the initial survey results, our intervention consisted of showing each patient instructional videos detailing the setup of low-vision accessibility features on their smartphone. Videos were available for both iPhone and Android users. In each case, the videos included instructions on configuring voice-over, magnification, and zoom functions and altering text size and contrast. The members of our research team wrote and recorded these videos, with smartphone instructions loosely based on the features detailed in the article “Tablet and Smartphone Accessibility Features in the Low Vision Rehabilitation” by Irvine et al. [12]. Finally, the patients repeated the same surveys either at follow-up visits or via phone calls.

The univariate analysis of pre- and post-intervention survey results was performed, with a significance threshold of p < 0.05. We used Fisher’s exact test to assess categorical variables and the Wilcoxon test for both ordinal and numerical variables.

## Results

Fifteen patients were recruited across six months. Thirteen (87%) used an Android phone, while two (13%) used an iPhone. The populations’ demographic features are included in Table 1.

**Table 1:** Study Population Demographics

	n	Percentage of the Study Population
Gender		
Male	10	67
Female	5	33
Age at the Time of Initial Survey Completion (Years)		
Median = 70		
50-60	5	33
60-70	2	13
70-80	7	47
80+	1	7
Patient Race		
White/Caucasian	9	60
Black or African American	6	40
Patient Ethnicity		
Not Hispanic, Latino, or Spanish Origin	15	100
Other Demographics		
Native Language English	15	100
Insured	15	100

The initial survey results of smartphone accessibility features showed that six (40.0%) of the patients used the text sizing/bolding tool at baseline. The remaining features, voice texting, zoom in/out, and magnifier, were each used by 27% of the study population at baseline, with a median value of one function used per participant. Thirteen (87%) participants indicated confidence in making adjustments for both phone calls and text messages at baseline, and this did not change following the instructional videos.

The patients completed the post-intervention survey at a mean of 44 days following their viewing of the instructional video (range: 15-102). Following our instructional videos, the median of accessibility features used increased from one to two (p = 0.11). The largest difference in pre- and post-survey results was found with the use of the magnifier tool, as 33% more participants reported using the feature following our intervention. Additionally, there was a non-statistically significant reduction in text messaging visual limitation recorded on the post-survey (p = 0.27). The data is shared in more detail in Table 2.

**Table 2:** Pre- and Post-survey Results of Accessibility Features, With Associated Comparative Statistics IQR: Interquartile Range

	Frequency or Median	Percentage or IQR	Frequency or Median	Percentage or IQR	
Variable	Pre-intervention (n = 15)		Post-intervention (n = 15)		P-value
Confidence in Adjusting Mobile Device Communication Medium (Assessed With Fischer’s Exact Test)					
Phone Call	13	86.7	13	86.7	1.00
Text Message	13	86.7	13	86.7	1.00
Mobile Device Function Use					
Voice Texting	4	26.7	5	33.3	1.00
Zoom In/Out	4	26.7	8	53.3	0.26
Magnifier	4	26.7	9	60.0	0.14
Text Sizing or Text Bolding Adjustment	6	40.0	8	53.3	0.72
Number of Functions Used	1	0-2	2	1-3	0.11
Visual Limitation on Mobile Device Communication Medium (Scales 1-5, Assessed With Wilcoxon Test)					
Phone Call	1	1-2	1	1-2	0.92
Text Message	2	1-3	1	1-2	0.27

At baseline, the median score in each domain of the EQ-5D-5L survey was 1. There was no significant change found in any of these domains following the implementation of our instructional videos. However, we did observe a non-statistically significant increase in median score of the general health score measurement. Additional detail is included in Table 3.

**Table 3:** Pre- and Post-survey EQ-5D-5L Results, With Associated Comparative Statistics IQR, Interquartile Range; EQ-5D-5L, EuroQol 5 Dimension 5 Level

	Median	IQR	Median	IQR	
Variable	Pre-intervention (n = 15)		Post-intervention (n = 15)		P-value
Self-Reported Health Scores (Assessed With Wilcoxon Test)					
Mobility (Scales 1-5)	1	1-2	1	1-2	0.71
Self-Care (Scales 1-5)	1	1-1	1	1-1	0.97
Usual Activities (Scales 1-5)	1	1-2	1	1-2	0.72
Pain and Discomfort Scale (Scales 1-5)	1	1-2	1	1-2	0.74
Anxiety and Depression (Scales 1-5)	1	1-3	1	1-2	0.91
General Health Score (Scales 1-100)	75	56-83	81	75-95	0.20

## Discussion

In this study, we sought to evaluate the impact of clinical education modules detailing low-vision accessibility features on patient quality of life and smartphone usage. We found no significant difference in the quality of life as measured by the EQ-5D-5L survey. While certain measurements in the smartphone usage survey demonstrated an increase following our video intervention, these observations did not reach statistical significance. Still, we conclude that providing educational material for enhanced smartphone usage has the potential to benefit patients with low vision, but further studies with larger sample sizes are needed to assess the significance of our findings.

Our smartphone instructional videos helped to address an important lack of clinical education on navigating technology in an accessible manner. While certainly not a replacement for the full spectrum of low-vision Services, our intervention represents a step in addressing the documented low rate of referral for low-vision rehabilitation [13]. Our videos may be used to introduce available options to patients with low vision while they await further referral. Previously, Irvine et al. developed an article compiling written instructions for the use of low-vision features found on smartphones [12]. This study provides a framework for introducing these tools to patients and their caregivers through easy-to-understand videos, with demonstrations given in real time.

Despite not reaching statistical significance, the patients reported using more of the accessibility functions on their smartphones and experienced a measured increase in quality-of-life measures. Because of this, we conclude that it is reasonable to include instructional videos in the list of resources available to patients seen in the glaucoma clinic. For example, providing a Quick Response (QR) code or link in their visit summary offers a possible increase in function with no additional risk. Likewise, future coordination with low-vision providers would be a beneficial next step. This would allow consistent, repeat education on smartphone setup and the tailoring of accessibility features to the patient’s unique needs.

The primary limitation of our study is the small sample size. Our data shows trends toward increased smartphone function, though it is possible that our limited sample was insufficient to detect statistical significance. Likewise, all 15 of our patients were native English speakers. Our instructional video would need to be re-recorded in other languages in order to meet the needs of non-English-speaking patients. Future studies could prolong the data collection period and broaden the inclusion criteria to both increase and diversify the patient population. Finally, future studies could employ the use of a quality-of-life metric designed to assess visual function in place of the EQ-5D-5L survey.

## Conclusions

Incorporating educational resources on the use of smartphone accessibility features may be accomplished in a risk-free, efficient manner through instructional videos. Because of this, it is worthwhile to provide these for patients with vision loss due to glaucoma. In doing so, one may expect an increased proficiency in the use of accessibility features. More studies utilizing a larger sample size and randomized control methodology are needed to further assess the impact on patient quality of life.

## References

[1] Martiniello N, Eisenbarth W, Lehane C, Johnson A, Wittich W (2022). Exploring the use of smartphones and tablets among people with visual impairments: are mainstream devices replacing the use of traditional visual aids?. Assist Technol.

[2] Robinson JL, Braimah Avery V, Chun R, Pusateri G, Jay WM (2017). Usage of accessibility options for the iPhone and iPad in a visually impaired population. Semin Ophthalmol.

[3] Lancioni GE, Singh NN (2022). Assistive technologies for people with diverse abilities. https://link.springer.com/book/10.1007/978-1-4899-8029-8.

[4] Owsley C, McGwin G Jr, Lee PP, Wasserman N, Searcey K (2009). Characteristics of low-vision rehabilitation services in the United States. Arch Ophthalmol.

[5] Freeman EE, Muñoz B, West SK, Jampel HD, Friedman DS (2008). Glaucoma and quality of life: the Salisbury Eye Evaluation. Ophthalmology.

[6] Nelson P, Aspinall P, Papasouliotis O, Worton B, O'Brien C (2003). Quality of life in glaucoma and its relationship with visual function. J Glaucoma.

[7] West SK, Munoz B, Rubin GS (1997). Function and visual impairment in a population-based study of older adults. The SEE project. Salisbury Eye Evaluation. Invest Ophthalmol Vis Sci.

[8] Kastner A, King AJ (2020). Advanced glaucoma at diagnosis: current perspectives. Eye (Lond).

[9] EuroQol Group (1990). EuroQol--a new facility for the measurement of health-related quality of life. Health Policy.

[10] Hernandez G, Garin O, Dima AL (2019). EuroQol (EQ-5D-5L) validity in assessing the quality of life in adults with asthma: cross-sectional study. J Med Internet Res.

[11] Jankowska A, Młyńczak K, Golicki D (2021). Validity of EQ-5D-5L health-related quality of life questionnaire in self-reported diabetes: evidence from a general population survey. Health Qual Life Outcomes.

[12] Irvine D, Zemke A, Pusateri G, Gerlach L, Chun R, Jay WM (2014). Tablet and smartphone accessibility features in the low vision rehabilitation. Neuroophthalmology.

[13] Kaleem MA, Swenor BK, West SK, Im L, Shepherd JD (2019). Low-vision services and the glaucoma patient. Ophthalmol Glaucoma.

